# A rapid evidence review of evaluation techniques for large language models in legal use cases: trends, gaps, and recommendations for future research

**DOI:** 10.1007/s00146-025-02741-9

**Published:** 2025-11-21

**Authors:** Joshua Kelsall, Xingwei Tan, Aislinn Bergin, Jiahong Chen, Maria Waheed, Tom Sorell, Rob Procter, Maria Liakata, Jenny Chim, Serene Chi

**Affiliations:** 1 University of Warwick, Coventry, UK; 2 University of Nottingham, Nottingham, UK; 3 University of Sheffield, Sheffield, UK; 4 Queen Mary University of London, London, UK

**Keywords:** AI and Law, Legal AI, AI benchmarking, AI Review, AI Metrics, Evaluation

## Abstract

The legal profession faces mounting pressures, including case backlogs and limited access to legal services. Large language models (LLMs), such as OpenAI’s GPT series, have been touted as potential solutions, promising to streamline tasks such as legal drafting, summarisation, analysis, and advice. Proponents argue these models can enhance efficiency, accuracy, and access to justice. However, significant risks remain. LLMs are prone to bias, factual hallucinations, and opaque reasoning processes, which can have severe consequences in high-stakes legal contexts. For responsible use in law, legal use cases must be accurately operationalised into LLM tasks that are sensitive to legal settings, as do the evaluation metrics used to evaluate LLMs performing those tasks. This paper presents a rapid literature review of LLM research in legal contexts since ChatGPT-4’s release in March 2023. We examine how legal tasks are operationalised for LLMs and what evaluation metrics are used, with a focus on how these align—or fail to align—with real-world legal practice. We argue that existing studies often overlook the institutional, organisational, and professional contexts in which these tools would be deployed. This oversight limits the practical relevance of current evaluations and proposes directions for more contextually grounded research and responsible deployment strategies.

**Supplementary Information:**

The online version contains supplementary material available at 10.1007/s00146-025-02741-9.

## Introduction

The legal profession faces unprecedented demands, with backlogs affecting many countries. Some claim large language models (LLMs) offer a solution by accelerating tasks including document drafting, summarisation, legal analysis, and legal advice (Dev 2024; Lightbody 2025; Schindler 2025). LLMs—such as OpenAI’s GPT series—use advanced natural language processing (NLP) to generate material that is claimed to be comparable to human output, boosting efficiency and accuracy. Others claim LLMs may enhance access to justice for those unable to afford traditional legal services (Chien and Kim 2025; Simshaw 2022; Steenhuis 2024).

However, LLMs pose significant risks. Bias, hallucinations, and legal misinterpretation can have serious ramifications for lawyers, judges, and the public. Poorly functioning LLMs can result in bad legal advice, unfair legal judgments, legal misunderstandings and misinformation. LLMs may fail to follow legal reasoning procedures, and their outputs may be opaque to lawyers, who need to explain them to clients or colleagues. There are data protection and information security risks. Lastly, law’s linguistic complexity means that LLMs insufficiently trained on legal data may struggle to interpret and generate legal texts. There have been some high-profile cases of failure, such as LLMs citing fictitious cases that lawyers have subsequently used in court, exposing lawyers to criminal charges (Tobin 2025).

Responsible deployment of AI requires two steps. First, legal use cases must be broken into corresponding tasks. For example, legal judgement prediction (LJP) may be broken down into a classification task to determine a guilty/not guilty verdict and a reasoning task to ensure the decision follows appropriate legal reasoning procedures. If tasks are insufficient for the use case, then the resulting technology may not work in real-world settings. Second, metrics must be used to evaluate LLMs—metrics that reflect actual user needs in real-world settings.

This paper provides a rapid literature review on LLM research since ChatGPT.4’s release in March 2023. We aim to understand both how legal use cases are interpreted as tasks performed by LLMs and the metrics and evaluation methodologies used to assess them. We examine this literature through a socio-technical lens, which draws attention to the role of non-technical factors in the study of technical innovations. These typically include, inter alia, work practices and workflows, professional standards, organisational norms and cultures (Hermann and Pfeiffer 2023; Uren and Edwards 2023). Based on our findings, we argue that an important gap exists in current research, in which generative AI systems are interpreted and evaluated without sufficient consideration of the legal contexts in which they would be deployed. We conclude with suggestions for closing this gap by improving the design of studies and the evaluation of LLMs in the legal domain.

## Methodology

Our search followed a rapid review two-step screening method,^1^ using a simplified search over a shorter timeframe. Given the fast pace of AI research, we aim to provide a snapshot of studies published from 2023—the year that ChatGPT-4 was launched. The review supports ongoing efforts to develop new benchmarks and metrics for evaluating LLMs in legal settings.

We conducted two searches on Scopus—one of the largest databases of peer-reviewed research. The first included studies published from 01/03/2023 to 13/05/2024. Our search terms were: “LLMs in law” | “Large Language Models in law” | “Large language models in legal use cases” | “evaluating LLMs in law”. It returned 101 records; 83 were retained after title and abstract screening. JK screened all, with a sample of 20 double-checked by JC, AB, and XT for agreement. The second SCOPUS search—using the same terms—was conducted on 11/02/2025 to account for papers published since our first search. This returned 150 studies. After removing duplicates and applying the exclusion criteria across both searches, 140 papers remained. Our full dataset is published on Mendeley (Kelsall et al. 2025).

Inclusion criteria included all English-language papers evaluating AI systems that use LLMs^2^ in legal settings, including quantitative and qualitative empirical studies, as well as theoretical studies. Exclusion criteria were studies that do not leverage LLMs as part of their AI systems, summaries of conferences, those not in English, or those focussing on domains other than law.

We recorded data on the legal domain/s studied, use cases, and tasks; the legal system of the country being studied; the LLMs studied in the paper; the evaluation methods and metrics; how the LLMs performed; and the proposed target groups of the use case (e.g. lawyers). In what follows, we focus on how use cases are broken down into tasks, and the metrics used to evaluate those tasks.
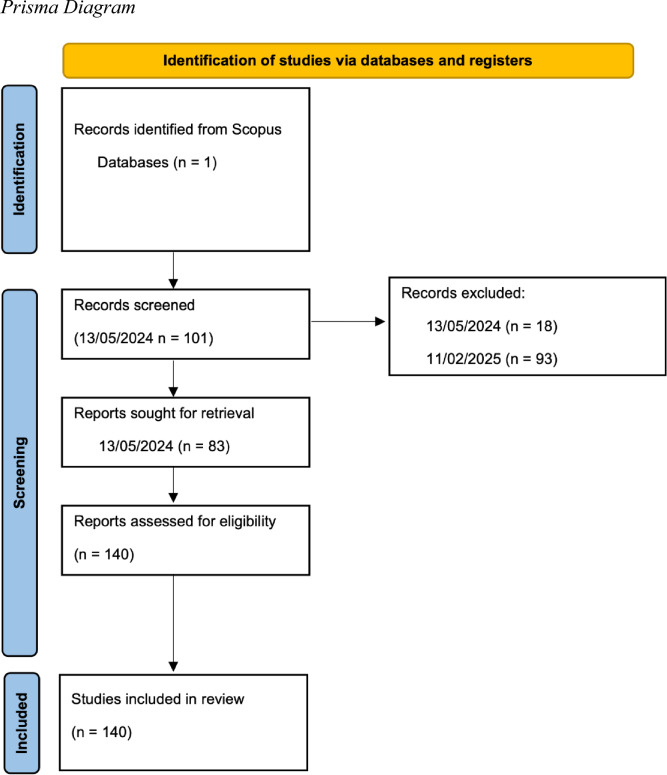


We adopt a socio-technical approach in our review. Technical evaluation often priorities quantitative benchmarks favouring functional reliability, often abstracted from deployment contexts (Baresi et al. 2024; Torkamann et al. 2024). In contrast, socio-technical analysis considers how technical systems function within real-world deployment settings.

We find that current research reveals a “socio-technical gap” (Ackerman 2000)—that is, a disconnect “between the human requirements in a technology deployment context [in this case, Law], and a given technical solution” (Liao and Xiao 2025). Such gaps arise because human activity is “highly flexible, nuanced, and contextualized”, while purely technical approaches use computational mechanisms that are “fragile and brittle” due to their formalisation which abstracts from the organisational and social contexts in which they are subsequently deployed (Liao and Xiao 2025: 1). Moreover, even considering the technical approaches to evaluation used in the papers we are reviewing these do not often follow the state-of-the-art in terms of metrics (e.g. accuracy used to assess generation tasks) and are therefore not fit for purpose. We conclude with recommendations for bridging this gap, aiming to improve evaluation methods and make AI tools more useful for legal professionals.

## Results

This section is organised into two main categories from our review. First, the interpretation of legal use cases as LLM-based tasks. Second, the metrics used to evaluate LLM performance on those tasks. To focus on dominant trends, we report only use cases and metrics appearing in at least five studies. Full results can be found in our database (Kelsall et al. 2025)

### Domain, use case, and task

We analysed LLM applications to law across three levels. The broadest is the legal domain, which refers to the legal domain of study, such as contract law or criminal law. Next is the use case, which is the general legal activity the LLM supports, such as legal judgement prediction (LJP), legal question/answer (QA) systems and legal document drafting. Finally, there are the NLP tasks the LLM performs to fulfil the use case, such as classification, information extraction, reasoning, text generation, summarisation, and retrieval.

Our review covered 46 legal domains. 10.7% of papers did not focus on specific legal domains—for instance, some QA systems tested LLMs’ ability to answer legal questions—often in the context of legal advice—without targeting defined legal domains (Janatian et al. 2023; Cesta 2024; Cheong et al. 2024; Long and Palmer 2024). We classified these as “general”. This could be problematic because legal and ethical codes differ across domains, and generalised models may miss domain-specific features. Among specified domains, contract law was the most common (10%), followed by criminal law (9.2%) and legal analysis (7.8%). Other notable domains include legal examinations (5%), human rights law (5.7%) and statutory law (4.2%). As these low percentages show, no domain dominated the research.

We categorised most use cases under four types:

1. *Legal analysis*: LMMs automate research-based activities including analysing legal documents such as contracts, assessing documents for compliance/violation detection, and analysing legal judgements.
2. *Legal document drafting*: LLMs automatically draft legal documents such as contracts or intervention proposals for legal mediation.
3. *Legal QA systems*: LLMs respond to questions, often as a legal advisor to laypersons.
4. *Legal judgement prediction*: LLMs predict judgements such as guilty/not guilty verdicts in court cases.

In line with our socio-technical approach, we analyse how each use case is broken into specific LLM tasks rather than listing tasks abstracted from use cases. This allows us to see whether the interpretations of use cases as tasks are appropriate. In what follows, we go through the major use cases and explain how they are broken down into LLM tasks. Then, in the discussion, we discuss the extent to which these task breakdowns are appropriate.

### Legal analysis

The most common use case was legal analysis (44%). Given its breadth as a category, we divided it into sub-use cases: legal document analysis (47%), legal compliance/violation detection (23%), and legal judgement analysis (21%).

#### Legal document analysis

In legal document analysis, LLMs examine official legal documents such as contracts (Savelka 2023; Zin et al. 2023), case law (Mumford et al. 2024; Prasad et al. 2024), statutes (Savelka & Ashley 2023), legal bills, and legal transcripts (Epps et al. 2023; Ramprasad et al. 2024).

Legal document analysis was typically broken down into classification and information retrieval tasks. For classification, LLMs were usually tasked with analysing documents and classifying their content within a set of pre-determined labels or categories (Table 1). For contract review, this consisted of entities including clause types (Iqbal 2023; Li et al. 2023; Savelka 2023; Savelka & Ashley 2023; Zin et al. 2023), party names, and dates (Zin et al. 2023).

**Table 1:** Legal document analysis tasks

Task	Total	Total (%)
Classification	12	19.35
Information extraction	9	14.51
Summarisation	5	8.06
Language modelling	1	1.61
Text generation	2	3.22
Information retrieval	2	3.22
Reasoning	1	1.61
N/A	2	3.22

Information extraction was a more complex task than classification. While LLMs could analyse legal documents to extract information relevant to a set of pre-determined categories, they could also perform the more complex task of identifying categories themselves. An example of the former, Zin et al. (2023) define a fixed set of queries such as “what is the agreement date?” “Who are the parties?” “What is the effective date?” and the LLM extracted information that answered these queries. As an example of the latter, Gray et al. (2024) tasked LLMs with reading court documents and identifying (ideally) legally significant factors which the LLM then used to categorise information. The models successfully produced rough factors that required further refinement by legal experts to ensure their relevance and applicability.

Information extraction was sometimes combined with other tasks, often as a basis for those tasks. For example, Iqbal (2023) aimed at assisting lawyers not only by identifying contract clauses, but by answering legal questions to provide insights for mergers and acquisitions. Contract analysis was broken into information extraction tasks, and then text generation and answer generation for the provision of advice to lawyers. Lam et al. (2023) utilised information extraction as a basis for contract drafting and refining legal clauses. However, such combination was rare. Although classification and information extraction are appropriate tasks for legal document analysis, they are often superficial and fall far short of the kind of in-depth analysis required for the full analysis of legal documents such as contracts, cases, and laws, especially in cases where there is a low tolerance for mistakes (Gray et al. 2024; Ma et al. 2023; Savelka 2023; Savelka & Ashley 2023).

#### Legal judgement analysis

For legal judgement analysis (LJA), LLMs explain legal judgements—either by analysing real court decisions and providing the underlying justifications, or as an additional component to LJP, where LLMs justify their own predictions by providing the reasoning that led to that prediction (Table 2).

**Table 2:** Legal judgement analysis tasks

Tasks	Total	Total (%)
Retrieval	1	7.6
Classification	7	53.8
Text generation	5	38.4
Summarisation	2	15.3
Reasoning	6	46.1
Event extraction	1	7.6
N/A	1	7.6

In 36% of the studies, LJA accompanied LJP to ensure the explainability of LLM predictions. In these cases, LJA was unanimously treated as a reasoning task. Huang and Ouyang (2023) focussed on generating coherent rationales by identifying causality and correlation between legal facts and charges. A key limitation of LLMs, such as LegalBERT, is that their decisions can be based on spurious correlations, in which unrelated facts are incorrectly linked to charges or rationales (Huang and Ouyang 2023; Geirhos et al. 2020). Addressing this, Huang and Quyang used counterfactual data generation to enhance the model’s causality reasoning capacities when generating judgements and their rationales. Vats et al. (2023) used chain-of-thought prompting to generate legal analysis of judgements, while Benedetto et al. (2024) used LLMs to generate explanations that selected the most relevant sentences from the judgement that most contributed to the predicted outcome. Here, LLMs were enhanced by annotating legal documents with legal entities (such as court names, petitioner names, and statutes) before processing, which improved both prediction and explainability and alignment with human explanations.

In studies not paired with LJP, LLMs analysed existing judgements. Sheik et al. (2024) used LLMs to identify overruling sentences, that is, whether a sentence represented an overruling or a non-overruling judgement (binary classification task); Drápal et al. (2023) used LLMs to thematically analyse court opinions, aiming to identify patterns and categories of theft from court decisions (multi-label classification task); and Al Zubaer et al. (2023) used LLMs to detect argument components in legal case judgements, such as premises and conclusions (multi-label classification task).

#### Legal compliance/violation detection

Legal compliance/violation (C/V) detection involves determining whether content in one or more documents complies with or violates requirements in others. This use case appeared primarily in auditing, but also in hate speech law and human rights law (Table 3).

**Table 3:** Legal compliance/violation detection tasks

Tasks	Total	Total (%)
Classification	9	53.2
Reasoning	5	35.7
Translation/semantic parsing	1	7.1
Text generation	1	7.1
Retrieval	1	7.1
Information extraction	1	7.1
N/A	1	7.1

In several studies (Baron et al. 2023; Chen et al. 2024; Luo et al. 2023), C/V detection was paired with LJP, where LLMs assessed violations or compliance based on legal text analysis. C/V detection was typically framed as binary classification, with LLMs providing yes/no answers regarding compliance or violation (Golgoon et al. 2024; Parizi et al. 2023; and Trozze et al. 2024). Exceptions to binary classification included, Berger et al. (2023), who used multi-label classification by including the categories ‘unclear’ or ‘not applicable’. Testing LLMs in the context of food safety regulations, Hassani (2024) used multi-label classification to reflect different relevant legal provisions. Baron et al. (2023) used classification but allowed for short written responses rather than consistent yes/no labels, which is due to the study including explanations of the C/V detection.

Accuracy in C/V detection requires aligning LLM outputs with the right regulations. Testing this in hate speech detection, Luo et al. (2023) trained LLMs on 11 definitions of hate speech drawn from three sources: the Canadian Criminal Code, Human Rights Code, and Hateful Conduct Policies collected from social media platforms. A multi-label classification task was used to determine whether an online post violated at least one of these definitions (yes/no/unclear). Reasoning was also used via the following prompt: “If Yes, explain why”. While the classification task achieved high performance—especially for non-fine-tuned GPT-4—the reasoning task decreased performance, with increasing hallucinations. The authors admit that the poor performance might be due to the zero-shot approach and simplistic prompting strategy. Reasoning seems to be the right task for ensuring LLMs’ decision-making conforms to legal requirements and reasoning techniques, though its results are mixed, dependent on prompting strategies, and not always aligned with legal reasoning.

### Legal QA system

The second most common use case was legal QA systems (31%), which use NLP and deep learning to answer legal question. QA systems were typically designed for laypersons seeking legal advice, though some were targeting legal professionals (Table 4).

**Table 4:** Legal QA system tasks

Task	Total	Total (%)
Long-form answer generation	23	58.1
Short-form answer generation	3	6.9
Classification	9	20.9
Reasoning	9	20.9
Information extraction	2	4.6
Regression	2	4.6
Retrieval	9	20.9
Summarisation	4	9.3
N/A	2	3.2

Legal QA was primarily framed as long-form answer generation tasks, often combined with reasoning—essential for ensuring explainability and alignment with legal advisory procedures and norms. Reasoning took various forms. Kang et al. (2023) tasked ChatGPT with legal reasoning following the IRAC (Issue, Rule, Application, Conclusion) method—a common reasoning strategy for legal professionals to structure legal analysis. Mavi et al. (2023) used chain-of-thought prompting for statutory reasoning about tax law and other financial questions. Janatian et al. (2023) tasked LLMs with extracting legal pathways from the Civil Code of Quebec by first extracting legal criteria and conclusions and representing how those legal rules lead to legal conclusions. Mavi et al. (2023) and Nguyen et al. (2023) tasked LLMs with generating Prolog code to demonstrate logical reasoning and rule-based logic in generating long-form answers. Yu et al. (2023) was based on the legal entailment task, a component of the Japanese bar exam, which tests a student’s ability to determine whether a given legal statement (hypothesis) is true or false based on specific legal premises.

Classification tasks were also common. Some studies posed multiple-choice legal exam questions. For example, Nay et al. (2024) tasked LLMs with answering multiple-choice questions on tax law, some requiring logical reasoning and mathematical calculations, although LLMs were not required to demonstrate their reasoning. Other studies tested classification in non-exam contexts. For example, working in the legal mediation domain, Tan et al. (2024) tasked LLMs with analysing text to classify appropriate intervention types, (multi-label classification), which informed text generation where the LLM-generated text based on the intervention type.

Retrieval and summarisation are also key tasks, both to ensure LLMs provide accurate and relevant legal information, documentation and citations, and that what is presented is readable and understandable to the relevant audiences. To this end, Hu et al. (2024) developed a retrieval system that responds to questions by retrieving relevant articles to the query, upon which it builds a short-form response. The combination of retrieval and text generation aims to ensure legal advice is based upon actual and relevant legal articles, which are imperative for sound legal advice.

### Legal judgement prediction

LJP was the third most common use case (17%). LJP involves predicting the outcome of legal cases based on case facts, precedents, statutes and other legal data (Table 5).

**Table 5:** Legal judgement prediction tasks

Task	Total	Total (%)
Classification	23	95.8
Text generation	5	20.8
Reasoning	6	25
Information retrieval	1	4.1
Short-form text generation	1	4.1

LJP was mostly reduced to classification, with few papers including reasoning or text generation as additional tasks. Typically binary, the classification task is to predict a verdict such as guilty or not guilty. While this is fundamental to predicting legal judgements, a handful of studies introduced more tasks. Jiang and Yang (2023) and Deng et al. (2023) used LLMs to generate detailed legal judgements in short paragraphs, which included a recommendation to the court that specifies the charges and relevant persons in the case and that demonstrated legal syllogistic reasoning. Benedetto et al. (2023), Vats et al. (2023) and Baron et al. (2023) also included reasoning tasks alongside LJP. Vat’s et al. used chain-of-thought prompting for reasoning while Benedetto et al. used LLMs to generate explanations that selected the most relevant sentences from the judgement that most contributed to the predicted outcomes. Baron et al. used a simple “explain why” prompt. Introducing these further tasks not only increases complexity but may be necessary insofar as legal judgements must be both explainable to judges and those they affect and must demonstrate alignment with legal practice.

### Legal document drafting

For legal document drafting, LLMs generate legal documents, ranging from contract clauses to entire contracts, interventions in the context of mediation, and summaries of legal documents including bills, transcripts and cases (Table 6).

**Table 6:** Legal document drafting tasks

Task	Total	Total (%)
Text generation	6	37.5
Long-form answer generation	2	12.5
N/A	3	18.7
Reasoning	2	12.5
Summarisation	5	31

Naturally, text-generation and summarisation are the primary tasks. Lam et al. (2023), and Iqbal (2023) were concerned with generating contract clauses, Calamo et al. (2023) used LLMs for drafting legal judgements, and Westermann et al. (2023) tasked LLMs with generating interventions in the context of meditation for landlord/tenant disputes. Westermann et al. aimed at using LLMs to resolve hostility in the mediation process, which is a very different focus from contract generation or legal judgement drafting. For mediation, correct analysis of hostility and the ability to use language to de-escalate situations is necessary, whereas legal reasoning is more important for legal judgement and contract drafting, as legal decisions and contracts may need to be explained or justified to laypeople and other legal professionals.

### Metrics

This section examines the metrics used to evaluate LMM tasks. Overall, 80.7% of the studies used quantitative metrics, 35.7% used qualitative evaluation, 21% used both, and 7.8% were theoretical papers.

#### Quantitative metrics

Among the quantitative metrics, the most common was F1 score (41.4%), followed by recall (32.1%), accuracy (30.7%), and precision (30%). Usage dropped off sharply after that, with only ROUGE (8.5%), BLEU (6.4%), and Exact Match (4.2%) used in at least five papers. In total, we identified 64 metrics, but most were included in only one paper. Some of these will be discussed in what follows; however, as they are exemplar metrics designed specifically for legal LMM evaluation.

F1 score, precision and recall were often but not always used together and used to evaluate models in classification tasks. Precision measures the proportion of correctly predicted positive instances out of all instances classified as positive. Recall measures the proportion of actual positive instances correctly identified by the model. F1 score is the harmonic mean of precision and recall (Goutte and Gaussier 2005). We define accuracy as a quantitative measure as we found it used for classification tasks where models are given a set of questions with pre-determined answers—as in legal examinations. Accuracy is measured by the number of correct answers provided by the model. A paradigm example is Katz et al. (2024), which tested ChatGPT against the multiple choice and long-form answers from the US bar examination mark scheme (Table 7).

**Table 7:** Quantitative metrics

Metric	Total	Total %
F1 score	59	41.1
Precision	42	30
Recall	45	32.1
Quantitative accuracy	43	30.7
ROUGE	12	8.5
BLEU	9	6.4
Exact match	6	4.2

These quantitative metrics are typically calculated by comparing LLM outputs to a ground-truth dataset—such as actual court rulings (for LJP), legal corpora (e.g. LEDGAR, CUAD, MAUD) or marking schemes for bar exams and legal QA competitions (e.g. COLIEE and ALQAC). However, since LLMs generate free-form text, mapping outputs to discrete labels for calculating precision, recall, F1 score and accuracy presents a significant challenge (Minaee et al. 2024; Barandoni et al. 2024; Harris et al. 2024). Most work uses hard-coded rules (e.g. exact matched and regular expressions) to find the matching text in the responses for determining whether the label is correctly identified. Such approaches rely on fixed rules and thus are susceptible to changes in the response (He et al. 2023; Lam et al. 2023; Savelka 2023; Terrón et al. 2023). Therefore, the metrics computed from these identified labels cannot reflect the correctness of the responses.

Although metrics such as ROUGE and BLEU were uncommon—accounting for just 8.5% and 6.4% of papers, respectively—it is worth highlighting Ammar et al. (2024), which found these metrics to be ill-suited for LJP, which is pertinent as 23% of LJP studies used at least one of these metrics. Ammar et al. used qualitative evaluation and quantitative BLEU and ROUGE scores for predicting court rulings based on actual court case descriptions from a dataset of 10,813 commercial court cases in Arabic. Since ROUGE and BLEU focus on exact matches rather than on capturing underlying textual meaning, low scores can result if outputs do not match ground truth. However, for LJP, what matters are accurate predictions that are semantically correct, regardless of word overlap. They found the qualitative evaluations to be more reliable in consequence, because human beings can recognise where text is semantically correct, even if there is minimal textual overlap between the output and ground truth.

Although Ammar et al. focus on LJP, the same point may apply to other use cases—including legal QA systems, legal document drafting and summarisation—where what matters is that the generated answers, documents, and summaries accurately capture the important legal meaning, rather than textually matching ground-truth documents. This is supported by Ma et al. (2023), who argue that legal documents produced by actual legal professionals are often heterogeneous in content, diverse in both style and opinions. To this end, metrics such as semantic matching, semantic similarity, and fuzzy matching may be appropriate, but were only used in a minority of studies (Azeem and Abualhaija 2024; Hamdani et al. 2024; Roegiest et al. 2023; Zhang et al. 2024). Hamdani et al. (2024) compared exact matching with fuzzy and alias matching and found that LLMs perform significantly better when measured by these metrics for answer generation for a legal QA system. Precision measured by exact matching produced a score of 35%, whereas it increased to 73% and 81.2% for alias and fuzzy matching, respectively (ibid: 8).

Reflecting these concerns about metrics, Liu et al. (2024) developed the legal text score (LTS) for legal summarisation. Based on general metrics such as BARTScore, LTS incorporates domain-specific knowledge by weighting key legal terms more heavily, which allows for paraphrasing of non-legal or unimportant terms. This helps ensure accurate yet readable outputs, especially in useful tasks such as legal QA, LJP and LJA.

LTS aligns with a socio-technical approach to evaluation by accounting for the importance of domain-sensitive language. Since the correct use of specialised domain language is necessary in legal domains, LTS reflects this in its weighting of its score. However, as a somewhat generalised metric, it may still lack sensitivity to different legal contexts. A more robust metric may need increased sensitivity to the specific needs of different legal domains, since the norms governing domains and their context of deployment will often differ. For example, when providing legal advice, a system must use non-legal language to communicate legal concepts in a way that laypeople can understand (without being misleading), while a summarizer working for a lawyer can be more jargon-heavy, as legal professionals are familiar with the technical language. Thus, even if a text is semantically correct and accurate for a lawyer, it may be poor quality for laypersons. Moreover, semantics is only one of the possible dimensions with factuality, evidence appropriateness, and coherence being other aspects to evaluate (Fabbri et al. 2021; Gehrmann et al. 2023; Song et al. 2024).

#### Qualitative evaluations

Qualitative evaluations varied in strategy and evaluator type. Of the 28 qualitative papers, 58% included author evaluations (typically legal academics), 34% used professional evaluations, and 16% used law student evaluations. Evaluators can make a big difference to the robustness of the findings. For legal use cases, using multiple evaluators would improve inter-evaluator reliability, and evaluations by legal professionals increase the domain sensitivity of the evaluations. Layperson evaluations are also valuable for use cases that affect them (e.g. for legal advice) to ensure that the advice responds to their needs effectively.

Aside from scalability, a worry with qualitative evaluations is the heterogeneity of expert evaluations. In their paper, Ma et al. (2023) tasked expert lawyers (mid-to-senior level) with reviewing and annotating contracts; specifically, by identifying conflicts between clauses and assessing interaction effects between provisions. They found significant heterogeneity in expert interpretations of contract clauses. Of 43 identified conflicts, only two participants had similar annotations. Overall, there was little agreement about which clauses conflicted or interacted, suggesting substantial variability in interpretation. This variability is worrying if studies fixate on LLM-generated output matching with a single—or even a small handful—of ground-truth documents or evaluators. It also further stresses the point that quantitative studies that compare outputs with a single ground-truth data set are unreliable forms of assessment, since it is possible for generative AI to generate outputs that, while not matching the ground-truth dataset, are still reliable and accurate responses (Novikova et al. 2017; Gehrmann et al. 2023), just as it is possible for different lawyers to produce outputs that are heterogeneous but still up to the appropriate legal standard. To this end, approaches that do not require comparisons against fixed ground-truth data, i.e. reference-free metrics, can help assess model-generated texts in a flexible manner. These approaches aim to capture intrinsic properties of the texts in their own right (e.g. coherence, conciseness) (Yuan et al. 2021; Xie et al. 2024) or in relation to source materials and knowledge bases (e.g. factuality) (Scialom et al. 2021; Min et al. 2023), and better accommodate variations that are afforded by the open-ended nature of generative AI, such as different topic foci and document lengths (Liu et al. 2023; An et al. 2024).

In most studies including legal practitioners, their role was limited to evaluating LLM outputs. While this is valuable, since practitioners have important domain knowledge, it is also important to include practitioners and other stakeholders in the design process. Cheong et al. (2024) exemplify this approach by studying expert perspectives about how LLMs ought to respond to laypersons’ legal queries. Rather than surveying individual opinions, participants were given cases composed of realistic legal queries surveyed from online forums and legal practices. In small groups, participants evaluated possible LLM response strategies ranging from refusing to answer to providing detailed legal advice. The result was a 4-dimensional framework outlining 25 key contextual factors that legal experts consider when evaluating the appropriateness of LLM-generated responses, including user traits, query types, AI limitations, and social consequences.

Similarly, Hagan (2024) focussed on laypersons in the context of AI legal advice. By getting laypersons to interact with Google’s Bard and then answering questions about their experiences, the study provides insights from the system users, which is arguably as important as expert evaluations for use cases where AI is deployed in a legal advisory capacity. Those unfamiliar with LLM prompting treated Bard like a search engine, with vague prompts such as ‘Tenant rights’, ‘Landlord issues’, and ‘Evicted by landlord’ (Hagan 2024: 9). This led to poor responses from the LLM. This research highlighted a fundamental problem with so-called prompt engineering strategies in which academics with AI experience create effective prompts. While researchers may create prompts that elicit high-quality outputs, if such prompting strategies are unavailable to those who use the system, then the system may be ineffective in practice.

Understanding how different users engage with legal AI helps clarify the real-world tasks LLMs must perform, what outputs are useful, and how those outputs should be evaluated.

## Discussion

This section discusses key themes emerging from our review of legal use cases, tasks, and evaluation metrics from a socio-technical perspective. Section 4.1 highlights the evaluation metrics, linking them to a benchmarking culture that prioritises general metrics over context-sensitive evaluation. Section 4.2 extends this critique to the socio-technical gap across the 140 studies, where complex legal use cases are oversimplified into tasks and detached from deployment contexts. Section 4.3 examines the lack of substantive ethical engagement and its implications for responsible research design.

### Benchmarking, metrics, and evaluation

Most of the surveyed papers relied on quantitative, reference-based, automatic metrics adapted from general NLP tasks. These metrics are ill-suited to evaluating LLMs in the legal use cases. We argue legal LLM research largely follows a benchmarking-focussed evaluation framework (Eriksson et al. 2025) which prioritises generalised, specifiable metrics over context-sensitive, qualitative assessments (Liao & Xiao 2025), and prefers static lab settings over real-world settings (McIntosh et al. 2024: 1–2).

Part of the reason for this is that benchmarking is generally seen as essential to AI development and evaluation. Eriksson et al. (2025: 2) note how “businesses go to great lengths to achieve good benchmarking scores… [sometimes spending] hundreds of thousands of dollars” to obtain high scores. Orr and Kang (2024) also note that benchmarking is deeply embedded in corporate marketing strategies and in increasing AI hype. Benchmarking is efficient and cheap, making it desirable in a research environment where there is intense publication pressure and being at the cutting edge of technological advances (Eriksson et al. 2025). Future research ought to focus on developing more meaningful metrics, and legal practitioners should be cautious of legal tech evaluated via abstracted metrics.

Cheng et al. (2025: 2–3) provide a useful overview of benchmarking critiques. These include data contamination, where public benchmarks leak into or are deliberately injected into training sets, leading to test-set memorisation and inflated scores (Dodge et al. 2019; Recht et al. 2019); cherry picking where benchmark creators collude with model creators to create hand-crafted suites that inadvertently or strategically favour certain AI models (Cheng et al. 2025); bias in test data (Phan et al. 2025); the devaluing of data collection and curation, such that data are often collected, reused and recycled without consideration of re-contextualisation for the new domain (Koch et al. 2021); simplified metrics that provide static snapshots of performance, and that generally only demonstrate task memorisation rather than true capability (Cheng et al. 2025; Wang et al. 2018).

We focus on the problem that most studies in our review adopt a benchmarking approach favouring generalised, quantitative metrics abstracted from real-world settings and do not even correspond to the state-of-the-art in quantitative evaluation for the tasks at hand. Accuracy, precision, recall and F1 score are commonly used metrics for evaluating classification tasks by comparing predicted labels directly against ground truth. However, this direct comparison is not applicable to generative LLMs. Generative LLMs generate free-form text, necessitating a post-processing step to map this text to discrete labels before evaluation. While some studies employ exact matching, others utilise fuzzy or semantic matching. Accurately mapping LLM-generated text to discrete labels can be challenging, as LLMs often paraphrase. Consequently, these metrics provide only an indirect measure of performance, potentially overestimating or underestimating capabilities depending on task complexity and the specific post-processing implementation. As we have discussed, there was some attempt to address these issues in the Liu et al. (2024) study which introduced the Legal Text Score, but the trend remains the application of generalised metrics that are ill-suited to the legal domain.

Metrics designed for generation tasks such as BLEU, ROUGE, and METEOR are also problematic. These metrics are based on n-gram matching, and while they have demonstrated reasonable correlation with human judgement in their original domains (ROUGE for summarization, BLEU and METEOR for translation), they have proven unreliable for LJP, Legal QA, legal reasoning and legal summarisation (Liu et al. 2024; Ammar et al. 2023). In these tasks, valid responses can be expressed using diverse phrasing, rendering n-gram matching against references a poor proxy for human evaluation. Furthermore, these metrics fail to account for the varying importance of different parts of a response. For instance, in LJP, the verdict carries the most weight. BLEU, ROUGE, and METEOR cannot adequately capture such nuances. Again, this suggests that law requires something like Lui’s Legal Text Score, as this score weights outputs in terms of the importance of different parts of a response.

Few papers conducted a critical analysis of the metrics used. As noted previously, one notable exception was Ammar et al. (2023), who combined quantitative and qualitative evaluation of predictions in the Saudi legal system. They found BLEU and ROUGE unreliable, as these n-gram-based metrics—designed for translation and summarisation—penalised predictions that differed in wording but semantically matched the ground truth. By contrast, human evaluators scored these outputs favourably, highlighting the mismatch between traditional metrics and legal reasoning. It is worth also noting that ROUGE and BLEU are metrics with well-known limitations for evaluation within the Natural Language Generation (NLG) community (Gehrmann et al. 2023). Such limitations include their inability to capture paraphrasing and terminological nuances (Dorr et al. 2005; Cohan and Goharian 2016), as well as low correlations with content quality (Reiter and Belz 2009) and real-world utility (Reiter 2018). Yet the identified works do not seem to be aware of these developments within NLG.

In Sect. 3.3, we showed that legal QA systems are often evaluating using legal exams like the UBE. While useful, passing such exams does not capture the broader skillset required for legal practice. As Kapoor et al. (2024) remark, “It’s not like it’s a lawyer’s job to answer bar exam questions all day”. Legal practice requires ethical judgement and contextual knowledge of the law, and one’s organisational policies that are not captured in standardized tests.

Henderson notes that, “while part of the licensing exam process might test with answering multiple-choice questions about what is written in the professional rules, it does not [test] a candidates ability to abide by them” (Henderson et al. 2024: 109). Another example highlights how lawyers also use knowledge about specific legal professionals and social dynamics. In one of our advisory board meetings for the AdSoLve project, participants working in the legal field noted that clients often wish to know information about the wider socio-legal environment. This involved personal information about the severity of judges, or the common strategies employed by the defendant’s lawyer in a court case. Such social and legal knowledge is not always written down, and is thus inaccessible to LLMs, which rely on textual information.

This limitation need not be overcome, but it is important to recognise the diverse skillset of lawyers, especially those skills that transcend their factual and text-based knowledge about the law. Recognising the limitations of LLMs and the broader skillset of lawyers helps define realistic expectations for LLMs and helps us better understand the place of LLMs in legal workflows.

Current benchmarking metrics contribute to the socio-technical gap by being too abstracted from deployment contexts, making them poor performance indicators in practice. Does this make such research irrelevant to legal practitioners? Not entirely. As McIntosh argues (2024) benchmarks can serve as an initial filter to exclude models that fail to meet basic competence. This “ensures that only LLMs with a foundational level of proficiency and regulatory compliance proceed to more rigorous evaluations, optimizing resource allocation for subsequent stages of the assessment process” (McIntosh et al. 2024: 14).

McIntosh also (2024) provides a potentially valuable strategy for ensuring benchmarks and evaluation metrics are contextually sensitive. This two-pronged approach evaluates benchmarks for functionality and integrity (McIntosh et al. 2024: 2). Functionality “refers to how well a benchmark measures the specific capabilities of an LLM in alignment with real-world application” while integrity “ensures that the benchmarks resist manipulation or gaming by models that exploit its criteria to produce misleading results” (ibid). Developing metrics that ensure functionality requires substantial research into deployment contexts to work out what good performance looks like, while integrity requires researchers to acknowledge the potential risks of their chosen benchmarks, such as data contamination when they reuse benchmarks and datasets.

Improving metric design also means broadening the range of expertise involved. Benchmarking needs to include quantitative metrics that follow the full range of aspects that correspond to requirements criteria set by users on the ground rather than generic metrics on generation. As Baresi et al. (2023: 2314) note, part of the reason for the bias towards functional correctness and reliability is that these are the metrics software developers themselves are most comfortable with. Work by Hagan (2024) and Cheong et al. (2024), which directly engages with stakeholder groups and utilises social science is a valuable way to improve metric selection. Other possibilities include conducting multi-disciplinary research in conjunction with social scientists, technologists, and philosophers.

### Large language models in law as socio-technical systems

Section 4.1 shows how benchmarking practices contribute to the socio-technical gap in studying LLMs in legal use cases. This section discusses how that gap persists in how legal use cases are interpreted as superficial tasks LLMs can perform.

Many studies reduced legal use cases—especially in LDA, LJP, and C/V Detection—into classification tasks. In some cases, these were binary classification tasks, as when LLMs predict a guilty/not guilty verdict for LJP or a compliance/violation verdict for C/V detection. In other cases, they were multi-classification tasks, as in Berger et al. (2023), where additional categories, such as “unclear”, were given, or where Zin et al. (2023) conduct legal document analysis in accordance with pre-defined labels.

Such simplification means that LLMs are applied only to surface-level, often pre-defined tasks. Indeed, recall that Gray et al. (2024) found that while LLMs were effective at extracting accurate and relevant information for LDA, further refinement required legal expertise. Whether this superficiality is useful to legal practitioners partly depends on whether the time that superficial analysis saves merits the cost of using AI systems. Regardless, these studies only support using LLMs for superficial tasks.

Binary and multi-classification tasks also fail to reflect the complexity of legal judgements and other decisions. Binary classification for LJP and C/V detection forces LLMs to provide concrete verdicts, even in cases that are ultimately ambiguous, which should be treated as such. Despite this, only a minority of studies included an “unclear” category,^3^ or allowed LLMs to provide a verdict as a text-generation task with an explanation.^4^ Thus, while classification may capture base-level accuracy and simple judgements, other tasks are required for LLMs to perform effectively and with depth.

The bias towards classification tasks is arguably a consequence of the benchmarking discussed previously. Metrics such as F1 score, precision, recall, ROUGE and BLEU are best suited to classification. Orr and Kang (2024: 1877) claim that benchmarks are normative instruments that perpetuate perspectives about how the world is ordered. In this case, benchmarks suited for classification encourage adopters of those benchmarks to frame legal use cases as classification tasks that are inadequate for the application domain, which requires more than mere baseline accuracy.

Another problem is that accurate classification or information extraction alone is often insufficient for legal tasks such as LJP, Legal QA systems for legal advice, and C/V detection. In these cases, LLMs must also explain their outputs in ways that align with domain-specific legal procedures and the intended audience’s expectations. Some studies attempted this by incorporating reasoning as a task to demonstrate explainability. This may mean moving away from classification altogether and favouring text-generation tasks which allow LLMs to demonstrate explainability and alignment with legal procedures.

In his work on developing ethical AI systems, Shin notes that a key problem with artificial intelligence is it cannot inherently feel or understand ethical values (2025: 4). In addition to this, the black box problem means that the outputs of AI are opaque to users, which is a problem as stakeholders, especially in legal contexts, must know the rationale behind critical decisions affecting their lives (Coeckelbergh 2020). Responding to this, some researchers try to develop “moral AI” in which machines are “taught to make ethical decisions by analyzying large datasets of human behaviour and ethical dilemmas” (Shin 2025: 5; Morley et al. 2023). Others argue that benchmarks are impossible to realise, and the focus should be values instead “shifting the emphasis from ethics to values gives rise to several new ways of understanding how researchers might move forward with a programme for robustly safe or beneficial AI” (LaCroix & Luccioni 2025: 2).

In the legal context, we claim that what matters is not so much ethical AI in Shin’s sense, but rather AI that is transparent in terms of its decision-making processes. Increasing the transparency of AI by making it both explainable and interpretable, and ensuring that its explanations are in line with legal practices allows for better scrutiny, which itself enables the ethical and responsible deployment of AI. This makes reasoning a critical task, particularly for legal QA systems, LJP, C/V detection and legal document analysis. Across some studies, reasoning was used to support legal explanation, argumentation, and transparency. However, reasoning was often understudied—especially in LJP and legal QA systems—in favour of simpler classification tasks.

Where reasoning is included, the kind of reasoning must be appropriate to the legal domain. Some studies tested reasoning using a general Chain-of-Thought (CoT) method to elicit reasoning from LLMs (Mavi et al. 2023; Vats et al. 2023; Yu et al. 2023). However, CoT has major flaws: it can rationalise false answers (Turpin et al. 2023), generate rationales that are not faithful to the models’ underlying processes (Tannery et al. 2024), and be vulnerable to manipulation by “backdoor attacks”. Traditionally, such attacks involve contaminating the training data or manipulating model parameters to make the model produce malicious outputs. However, Xiang et al. (2024) proposed “BadChain” a backdoor attack against LLMs that uses CoT prompting, which bypasses training data access and therefore lowers the barrier to attack.

These risks matter in legal use cases, especially for legal QA systems for legal advice, where poor prompting can yield misleading legal advice, potentially causing serious legal harm to laypersons. The same concerns apply if legal professionals use LLMs to justify rulings or arguments without robust reasoning capabilities. This point is pertinent for when LLMs are not tasked to conform to specific forms of legal reasoning, as in Luo et al. (2023), where a simple prompt such as “if yes, explain why” is given.

In general, LLMs have been found to fail to support the complex tasks required for full legal reasoning (Dougrez et al. 2025). In part, this is because LLMs do not always align with the reasoning processes of legal professionals. In our review, we did find some studies that did attempt to align LLMs’ reasoning with legal reasoning. Indeed, some studies tested LLMs’ ability to conduct Issue, Rule, Application, Conclusion (IRAC) reasoning (Kang et al. 2023; Trozze et al. 2024; Yu et al.. 2023)—a standard structure used by trainee lawyers to structure legal arguments. While this is a step forward, and while reasoning tasks are more advanced than classification, they still fall short as the realities of legal reasoning cannot always be reduced to simple rules of thumb or procedures. Performance on IRAC does not mean models can handle other forms of reasoning, such as abstractive reasoning, which is integral to legal work. Indeed, Dougrez et al. (2025) and Nguyen et al. (2024) focussed on the capacity of state-of-the-art models used in legal reasoning to support abductive reasoning and found that SOTA models fell short.

The problem with task selection across the studies is that it is often superficial and detached from deployment contexts. Even with reasoning, the use of generalised chain-of-thought prompting is inappropriate for legal use cases since chain-of-thought prompting does not require LLMs to demonstrate legal reasoning processes. To improve task selection in future research, we suggest putting greater focus on understanding the intricacies of different legal use cases by greater collaboration with legal professionals. Doing so will elucidate the scope of tasks which LLMs need to perform to be responsibly deployed in context.

One way to do this is to consider the level of research realism of the study. Research realism refers to the closeness in context between the environment in which research is conducted and the environment in which it is applied (Liao and Xiao 2025: 3). Controlled studies in static environments using abstracted metrics like F1 score demonstrate a low level of research realism because the research context—in a static, controlled environment—is far removed from the deployment environment, which is not static, controlled, and often involves legal professionals and laypeople who may not be competent in using and understanding legal AI.

Future studies should aim for higher realism. This could involve collaboration with legal firms to test in sandbox environments to measure LLMs’ performance in practice, but also by engaging with legal practitioners to understand their ethical frameworks, workflows, and needs. A good example of a study from our review that takes the deployment context in mind is Iqbal’s (2023) study, which frames its selection of LLMs in terms of data control and privacy protection. Iqbal (2023) considered the consumer posture of the legal organisation when designing their LLM for a legal contract drafting use case. Consumer posture refers to the procurement of AI by a legal company, and ranges from a fully in-house developed model which ensures maximum privacy protection and data control, to fully outsourcing the AI tool using something like ChatGPT, which poses the biggest risk to privacy and data control. Iqbal used an LLM that matched the most appropriate consumer posture for practising lawyers, and thus, is another example of a study which takes into account the needs and ethical concerns of lawyers and clients when designing an experiment for this use case.

If Iqbal is right, testing GPT-style models may not be useful to many legal organisations, regardless of performance, because such models do not meet legal institutions’ privacy or data control standards. This underscores the value of engaging with legal professionals before conducting research, ensuring selected models are not just capable but also appropriate and aligned with legal practice.

Another way to improve realism is through direct engagement with stakeholders. In this vein, recall the studies by Cheong et al. (2024), and Hagan (2024), mentioned in Sect. 3.6.2. Cheong et al. focussed on understanding expert perspectives about how LLMs ought to respond to laypersons’ legal queries, while Hagan focussed on how laypersons engage with LLMs for legal advice. Such research helps identify the tasks LLMs need to perform in practice by grounding them in real deployment contexts, and also highlights the potential practical risks and ethical concerns that need to be addressed for responsible as well as competent use.

In workshops with leading UK law firms, we also uncovered important contextual features researchers ought to consider when selecting use cases. One presentation outlined the effectiveness of LLMs in different legal use cases, in conjunction with the amount of time that lawyers traditionally spend on this work. By highlighting this, researchers can focus on legal use cases that are both time-consuming and well suited to automation, rather than on low-impact tasks.

Engagement should also extend to the legal and regulatory frameworks governing each use case. Henderson argues that.

“Professional codes of conduct and rules can guide machine learning researchers to address potential gaps in benchmark construction. These guidelines frequently account for situations professionals may encounter and must handle with care.,. for example, while part of the licencing exam process might test being able to answer multiple choice questions about what is written in the professional rules, it does not [test] a candidates ability to abide by them” (Henderson et al. 2024: 109).

Legal policies and professional standards vary across legal jurisdictions, domains, and organisations. An LLM that performs well on LJP in criminal law may not generalise to other areas. If LJP in criminal law requires decision-makers to follow certain reasoning procedures, then LLMs must be tested on those exact capabilities. Doing so gives legal practitioners greater assurance that legal tech does not violate their policies and codes of conduct.

A final challenge is that some legal tasks may resist formalisation altogether. Polanyi’s (1962) notion of tacit knowledge highlights that some practitioner expertise cannot be articulated in language. As we noted previously, giving legal advice to laypersons often requires context-specific knowledge about judges, opposing counsel, and the broader socio-legal environment. It may also demand emotional and cultural sensitivity, traits which were ignored outside of the studies that focussed on legal mediation interventions. Sensitivity to possible tacit knowledge is important, since it reminds us that however we might interpret legal use cases as specifiable tasks, there may always be elements to those use cases which are not fully captured by those tasks, thus making human interventions and collaboration with AI systems of primary importance.

In sum, then, current benchmarking and metric evaluations, as well as the ways that LLM use cases are broken into tasks, reveal a socio-technical gap in legal AI research. Filling this gap means designing benchmarks that reflect the real-world contexts of AI use. Researchers can work towards this by aiming for closer and more sustained engagement with affected stakeholders. Doing so will provide a better degree of research realism, which is increasingly necessary to understand how well LLMs perform legal tasks in practice.

### Integrating ethics into research: fairness and privacy

This paper has focussed primarily on task and metric selection for evaluating LLMs in legal use cases. However, socio-technical evaluation also requires attention to ethical concerns specific to each context. Therefore, this section outlines how future research can better integrate context-sensitive ethical considerations into the design of studies.

Our review found most studies lacked substantive ethical analysis, and very few integrated ethical considerations into their experiments. Consideration of ethical issues arising from the application of LLMs to legal use cases was minimal, often relegated to generalised discussions in “Ethical Considerations” sections which reiterate but do not suggest solutions to well-known ethical worries such as bias, privacy and trustworthiness. Most studies did not embed these ethical concerns in the tasks or evaluation metrics. This is problematic, as consideration of ethical issues like fairness and transparency is not enough; such ethical principles must be translated into actionable practices (Shin 2025).

To work towards this aim, we highlight two papers that demonstrated this kind of translation of ethics into practice. They serve as exemplars for future research to find ways of embedding ethical considerations more deeply in one’s research that are specific to the legal domain. These papers focus on operationalising fairness.

Malic et al. (2023) made the problem of unfair bias in legal judgement prediction the focus of their task. They tasked the LLM with predicting the racial category of different people involved in different criminal cases. This was done as a binary classification task with (black/white) as the two labels. The LLM was considered racially blind if it had a 50/50% chance of classifying a masked racial term as black or white. Though a robust task along these lines would have to account for a more varied class of racial categories, as well as other relevant social disparities, the study shows how an ethical consideration can be integrated into a legal use case and task and how it might be measured and evaluated.

In a similar article, but focussed on social disparities in India, Tripathi et al. (2024) offer a more formalised metric—the Legal Safety Score—to evaluate both the accuracy and fairness of statutory legal reasoning. The metric works by computing the weighted harmonic mean between the model’s F1 score (getting a legal prediction correct in a binary classification task), and its relative fairness score, which measures the consistency of an LLM in producing similar results across socially sensitive identity groups including caste, religion, and gender. Tripathi et al.’s metric is more complex than the one in Malic et al.’s both because it attempts to combine accuracy and fairness, and because it can account for multiple social groups.

Highlighting these studies allows us to demonstrate important issues concerning fairness. First is that what fairness looks like differs depending on the location of the study. Malic’s study focussing on racism towards African Americans may well be appropriate to a US context where that group faces prejudice in the legal system, but is less appropriate in the Indian context, where issues such as the caste system are relevant. It is important that when researchers design fairness metrics, they are aware of the contexts in which their research is relevant, as such norms may not translate or be relevant cross-culturally (Dhole 2023: 8; Dickerson 2020). Second, Tripathi’s study shows how fairness metrics must be weighed against accuracy metrics, especially important in legal contexts where LLMs may be tasked with providing information or making judgements. It is for this very reason that Tripathi offers the more complex legal safety score which weighs up accuracy with fairness, aiming to provide an appropriate balance between the two.

Of course, there are issues with the mathematical approach to defining fairness in these studies. As Dhole notes, definitions of fairness often “include procedural, contextual, and contested aspects that might not be resolved through mathematical formulation” (2022: 8). While we may be able to make AI less biased through something like a Legal Safety Score, it may be that ultimately, the ethical considerations remain the responsibility of human beings who play a key role in detecting the bias of systems. Indeed, Shin suggests users of such systems employ a “doubt heuristic” in which they approach AI recommendations with caution, especially in the criminal justice domain, where “biased AI decisions can have severe consequences” (Shin 2025: 21).

In this vein, we suggest that while attempting to make AI ethical is undoubtedly important, it is equally important not to outsource ethical thinking to AI. Users should remain aware of the potential ethical limitations of AI when relying upon it and adopting a doubt heuristic is one way they can do this. Furthermore, to better integrate ethics into future studies, greater collaboration is needed both with stakeholders but across disciplines, especially with ethicists from social science and philosophy.

## Conclusions and future work

Current research on LLMs in legal use cases in both task selection and evaluation has several shortcomings. Current benchmarking frameworks favour static research environments and quantitative metrics that are abstracted from real-world settings. Many studies do not involve legal professionals or stakeholders during design, resulting in research that is less applicable to real-world settings. Task selection is often superficial, geared towards quantitative metrics, and also abstracted from the complexity of deployment contexts. For example, the focus on classification tasks and general reasoning frameworks, such as Chain-of-Thought (CoT) prompting, limits the ability of these studies to capture the complexity of legal reasoning. Much of the research is “output focused” emphasising performance metrics over realistic and context-sensitive experimental design. Lastly, ethical issues such as bias, fairness, privacy, and trustworthiness are often mentioned but rarely embedded in the studies.

We conclude with recommendations for improving task selection, study design, and evaluation of LLMs in legal settings. These are aimed at researchers to help them make their work more useful to potential users, but they can also help stakeholders identify more rigorous evaluations of LLM tools.

1. Stakeholder-informed task development:

- Engage with legal professionals and all affected stakeholders from the beginning of the research to ensure tasks reflect real-world legal use cases. Use surveys, workshops, and discussions with legal practitioners and their clients.

- Improve prompting methods, particularly for legal QA systems, so that lay users can achieve accurate and actionable results. This can be done by engaging with these groups to see how they prompt LLMs, and what they expect to get out of these interactions.

2. Ethical and practical considerations in model design:

- Integrate fairness directly into tasks to mitigate biases, as seen in studies such as Malic et al. (2023) and Tripathi et al. (2024) that operationalised different kinds of fairness in legal judgement predictions.

- Ensure fairness reflects local legal and cultural contexts. Different regions and firms have different ethical standards that models must meet.

- Develop solutions that address legal firms’ privacy and security concerns, such as Iqbal’s (2023) proposed Creator Customiser model. This means testing LLMs that would be used in practice, even if this means creating bespoke models with greater privacy protection and customisation options than general-purpose LLMs like GPT.

3. Avoid narrow task evaluation:

- Recognise that strong performance on isolated, superficial legal tasks does not necessarily indicate overall suitability. This is due to limitations in the text-based knowledge on which LLMs are trained, and because of the influence of tacit knowledge in legal institutions and practices.

- Investigate task interdependencies—i.e. whether multiple interrelated tasks must be solved together for an LLM to be effective in a legal use case.

4. Developing robust evaluation metrics:

- Move beyond standard NLP metrics such as BLEU, ROUGE, and METEOR, which miss key aspects of legal accuracy, text generation, and reasoning.

- Encourage and conduct studies that are embedded in the relevant legal context. This means the legal domain, country/countries, and types of law firms (large vs small). Doing this will provide more concrete and reliable indicators of how well LLMs perform in their real-world contexts of use.

- Incorporate legal domain-specific evaluation methodologies that emphasise contextual accuracy and reasoning alignment with professional legal standards.

- Use mixed-methods approaches, combining quantitative metrics with qualitative human assessments, as exemplified by Ammar et al. (2023), to more accurately gauge LLMs’ legal performance.

- Build domain-relative evaluation metrics, as exemplified by Lui et al.’s (2024) Legal Text Score, and Tripathi et al.’s (2024) Legal Safety Score. Compare these against more traditional metrics as exemplified by Hamandi et al., (2024), to further understand the limitations of traditional metrics.

- Ensure that the domain-relativity is not just about the legal domain, but also the jurisdiction, and location. Ensure that it reflects legal, moral, and cultural norms and needs as well, e.g. see how Tripathi’s Legal Safety Score is embedded in Indian cultural prejudices, rather than Western ones, due to its Indian context.

## Limitations

A rapid review, although lacking the rigour of a systematic review, is an established methodology for capturing rapidly developing and early-stage research. However, its benefits present certain limitations. Using only one database may have excluded relevant legal or interdisciplinary papers. Still, reviewing 140 papers provided enough data for strong, if not fully generalisable findings. While rapid reviews are less rigorous and wide-ranging than systematic reviews, we implemented some systematic processes, such as involving multiple reviewers during screening, to address potential issues at this stage. Given the rapid pace of LLM research, this review offers only a partial snapshot of the current research landscape. Nevertheless, we believe our findings are sufficiently important to warrant publication, as they can serve as a foundation for future research.

Furthermore, due to the brevity of this rapid review, we were unable to present our data in a more accessible manner for non-legal and non-computer science professionals. Consequently, certain areas of the review may lack clarity for experts in other domains. However, we believe that the findings have been presented in a manner that is both useful and usable from an interdisciplinary perspective, and our full dataset is publicly accessible. It is worth noting that most studies primarily focus on prototypes and other early-stage research, which restricts the hypotheses we can draw about the real-world deployment of these technologies.

Lastly, our research was limited to papers in English. While the legal jurisdiction of the papers was wide ranging, including papers from China and India as well as Europe and America, our search may still overrepresent English-speaking countries and legal systems that utilise English. We therefore welcome and encourage future research that engages more broadly with non-English or multi-language jurisdictions and contexts.

## Supplementary Information

Below is the link to the electronic supplementary material.

Supplementary file1 (DOCX 245 KB)

## Data Availability

The Mendelay dataset can be found here: Kelsall, Joshua; Bergin, Aislinn; Chen, Jiahong; Tan, Xingwei; Waheed, Maria; Sorell, Tom; Procter, Rob; Liakata, Maria (2025), “Evaluating Large Language Models in Legal Use Cases”, Mendeley Data, V1, 10.17632/jnztrkb4f2.1 However, we have included the data repository file as part of the "related files" section of this submission, so it can also be accessed there.

## Footnotes

1 Grant & Booth (2009); Tricco et al. (2015); Tricco et al. (2017).

2 Although our focus in this paper is on LLM technology, this would apply to any use of artificial intelligence in a practical domain. It is necessary to ensure that when evaluating the performance of such technology, that they capture and evaluate the relevant dimensions of the use cases.

3 See Berger et al. (2023).

4 Baron et al. (2023).
